# Modelled Economic Analysis for Dacomitinib–A Cost Effectiveness Analysis in Treating Patients With EGFR-Mutation-Positive Non-Small Cell Lung Cancer in China

**DOI:** 10.3389/fonc.2021.564234

**Published:** 2021-12-14

**Authors:** Yong-feng Yu, Luan Luan, Fan-fan Zhu, Peng Dong, Li-Heng Ma, Lan-ting Li, Lan Gao, Shun Lu

**Affiliations:** ^1^ Shanghai Lung Cancer Center, Shanghai Chest Hospital, Shanghai Jiao Tong University, Shanghai, China; ^2^ Health Economics and Outcome Research, Pfizer Investment Co., Ltd., Beijing, China; ^3^ Department of Medical Affairs, Pfizer Investment Co., Ltd., Shanghai, China; ^4^ Shanghai PalanDataRx Co., Ltd., Shanghai, China; ^5^ Deakin Health Economics, Institute for Health Transformation, Deakin University, Geelong, VIC, Australia

**Keywords:** epidermal growth factor receptor (EGFR) mutations, NSCLC, cost-effectiveness analysis (CEA), partitioned survival analysis, economic model

## Abstract

**Objectives:**

To establish the cost-effectiveness of dacomitinib compared to gefitinib from the Chinese healthcare system perspective.

**Patients:**

Advanced non-small cell lung cancer (NSCLC) harbouring epidermal growth factor receptor (EGFR) mutations.

**Methods:**

Partitioned survival analysis was undertaken to examine the cost-effectiveness of dacomitinib utilising individual patient data (IPD) from the pivotal randomised controlled trial (RCT) (ARCHER 1050). The three health states modelled were progression-free, post-progression, and death. Parametric survival distributions were fitted to IPD against the Kaplan-Meier survival curves corresponding to progression-free survival (PFS) and overall survival (OS) outcomes by randomised groups. Costs included drug acquisition and administration, outpatient management (outpatient consultation and examinations), and best supportive care costs. Utility weights were sourced from the pivotal trial and other published literature. The incremental cost-effectiveness ratio (ICER) was calculated with costs and quality-adjusted life years (QALYs) discounted at an annual rate of 5%. Both deterministic and probabilistic sensitivity analyses were undertaken.

**Results:**

In the base case, dacomitinib (CNY 265,512 and 1.95 QALY) was associated with higher costs and QALY gains compared to gefitinib (CNY 247,048 and 1.61 QALYs), resulting in an ICER of CNY 58,947/QALY. Using the empirical WTP/QALY threshold, dacomitinib is a cost-effective treatment strategy for patients with EGFR-mutation-positive advanced NSCLC. The probabilistic sensitivity analysis suggested that dacomitinib had a 97% probability of being cost-effective.

**Conclusions:**

Dacomitinib is a cost-effective treatment strategy in treating patients with EGFR-mutation-positive NSCLC from the Chinese healthcare system perspective. The uncertainty around the cost-effectiveness of dacomitinib could be reduced if long-term survival data become available.

**Clinical Trial Registration:**

NCT01024413

## Introduction

With more than 2.1 million new cases and 11.6% of the total cancer incidence in 2018, lung cancer is the leading cause of cancer-related mortality (1.8 million deaths, 18.4% of the total) worldwide (1). Among these patients, more than a third of all newly diagnosed lung cancers were from China, constituting a substantial burden for patients, families, and society as a whole (2). In 2014, the China annual cancer report revealed that there were 782,000 patients with newly identified lung cancer (3), including 521,000 male and 261,000 female patients, which represented a significant increase (20%) from 651,053 total new cases in 2011 (4). It was estimated that the total national medical cost attributable to lung cancer was US$10.31 billion, accounting for 2% of the total medical cost in China in 2015 (5).

Of all lung cancer cases, approximately 85% are non-small cell lung cancer (NSCLC), and the majority of these patients are diagnosed at the advanced or metastatic stage, losing their opportunities for surgery. Epidermal growth factor receptor (EGFR) mutations are observed in approximately 50% of Asian and 20% of non-Asian patients (6). EGFR mutations occurred more frequently in patients who had never smoked, women, adenocarcinomas, and Asian patients (7–9).

First-line treatment options of NSCLC patients harbouring EGFR-mutation include the EGFR tyrosine kinase inhibitors (TKIs), gefitinib, erlotinib, and afatinib all demonstrating improvements in progression-free survival (PFS) and quality of life, compared with platinum-based doublet chemotherapy. There are approved second (treating NSCLC harbouring activating EGFR mutations) and third (i.e., osimertinib, targeting NSCLC carrying EGFR-TKI–sensitising and EGFR p.Thr790Met (T790M) resistance mutations) (10) generation EGFT-TKIs in China. Dacomitinib is a second-generation, irreversible EGFR TKI that was approved in China in 2019. It is a pan-HER irreversible inhibitor that has activity against all three kinase-active members of the ErbB family (EGFR/HER1, HER2, and HER4). The FDA and China Food and Drug Administration (CFDA) granted dacomitinib market access based on a randomised, multicentre, open-label trial (ARCHER 1050). This trial evaluated the efficacy and safety of dacomitinib versus gefitinib as a first-line therapy in patients with advanced EGFR-mutation-positive NSCLC. The outcome from this phase III trial showed that dacomitinib significantly improved PFS compared to gefitinib in first-line treatment of patients with EGFR-mutation-positive NSCLC, with median PFS of 14.7 vs 9.2 months, respectively (hazard ratio 0.59, 95% confidence interval, CI: 0.47-0.74, p<0.0001). also showed clinically meaningful improvement in overall survival (OS) with dacomitinib (11).

The National Drug Reimbursement List (NDRL) has four first-line EGFR TKIs (i.e., gefitinib, erlotinib, icotinib, and afatinib) currently registered to treat patients with EGFR-mutation-positive NSCLC dating back to 2016. However, given that the marked gap in the health outcome for patients with EGFR-mutation-positive NSCLC still exists and the availability of more effective treatment options, the next critical question to address is whether more effective treatment (i.e., dacomitinib) represents value-for-money, in other words, whether the increased benefits justify the increased costs. This is pivotal for the Chinese government since there is always a constraint between ever-increasing healthcare demand and the already stretched healthcare budget. In response, we aimed to undertake a modelled economic evaluation of dacomitinib in treating patients with EGFR-mutation-positive NSCLC from the Chinese healthcare system perspective using the ARCHER 1050 trial and local costing data.

## Methods

### Model Structure

Partitioned survival analysis was utilised to model the long-term cost-effectiveness of dacomitinib versus gefitinib. A proportion of patients can move among progression-free (PF), post-progression (PP), and death states. The progressed patient cannot return to the PF health state. This modelling approach was chosen because it is most widely used to summarise the overall impact of treatments on survival and health-related quality of life (HRQoL) in the context of clinical trials (12–15). The survival curves of progression-free survival (PFS) and overall survival (OS) were used independently to derive the proportion of the cohort at PF and PP (i.e., the difference in survival at the same timepoint from PFS and OS curves) health states by various timepoints. Thus, the proportion of patients in each modelled health state are time-dependent.

### Population

Patients diagnosed with EGFR-mutation-positive advanced NSCLC (stages IIIB/IV or recurrent) and at least one documented EGFR mutation (exon 19 deletion or the Leu858Arg mutation, with or without the Thr790Met mutation) were modelled. The baseline characteristics were defined as per the published clinical trial. Briefly, the modelled cohort had a median age of 62 years, with female participants overrepresented (>50%) and predominantly stage IV cancer (81%). Exon 19 deletion (59%) and Leu858Arg (41%) are the key EGFR mutation types.

### Long-Term Extrapolation

Treatment-specific PFS and OS curves from the pivotal trial were used to track the proportion of patients who stayed in the PF, PP, and death health states. Since the median duration of follow-up was 22.1 versus 23.0 months in the dacomitinib and gefitinib-treated patients, respectively, extrapolation of survival curves observed from the trial is necessary to assess the long-term cost-effectiveness of dacomitinib.

The ARCHER 1050 patient-level data were analysed to generate the within-trial Kaplan-Meier survival curves for PFS (assessed by the independent review committee) and OS by randomised groups. The recommended parametric survival distributions, including exponential, Weibull, log-normal, log-logistic, generalised-gamma, and Gompertz, were fitted to the within-trial Kaplan-Meier curves (16). The best fit curve for long-term extrapolation was selected based on the goodness-of-fit statistics (AIC and BIC values), visual inspection (17), and clinical validation. Input from clinical experts was sought to assess the plausibility of the extrapolation.

### Treatment Protocol

Hypothetical patients started either dacomitinib or gefitinib treatment in the first cycle of the partitioned survival analysis (PartSA) model. It was assumed that patients only discontinued the dacomitinib/gefitinib treatment (i.e., first-line treatment) upon disease progression (i.e., transition from PF to PP state). Those who progressed are eligible for second- and third-line treatment incorporating gefitinib, erlotinib, afatinib, osimertinib, and other standard chemotherapy (i.e., pemetrexed, and platinum-based chemotherapy). Around 71% of patients underwent the second-line treatment since the disease progression, and a further 48% of them received the third-line anti-cancer treatment. The duration of second-, third-, and subsequent treatment are summarised in **Supplementary Table 1**. The dosing regimen for each treatment is supplied in **Table 1**.

**Table 1:** Unit cost of healthcare resources included in the analysis.

Treatment	Unit price	Dosing regimen	Frequency per 28-day	Cost per cycle	Reference
**First-line**					
Dacomitinib	¥88/15 mg	45 mg/day	1	¥7,418	Local charge
Gefitinib	¥236/25mg	25 mg/day	1	¥6,608	Online resource (16)
**Second- & third-line**					
Erlotinib	¥195/150mg	150mg/day	1	¥5,460	Online resource (16)
Afatinib	¥200/40mg	40mg/day	1	¥5,600	Online resource (16)
Osimertinib	¥510/80mg	80mg/day	1	¥14,280	Online resource (16)
Docetaxel^*^	¥97/20 mg	120 mg	1.33	¥5082.40	Zeng et al 2012 (17)
Pemetrexed^^^	¥321/200 mg	850 mg	1.33	¥11212.71	Zeng et al 2012 (17)
Platinum-based therapy			1.33	¥18,174.39	Zeng et al 2012 (17)
Docetaxel+ platinum-based	–	120 mg+ 120 mg	1.33	¥13,282.55	Zeng et al 2012 (17)
**Chemotherapy drug**					
Cisplatin	¥64.31	128 mg	1.33	¥10,932.70	Zeng et al 2012 (17)
Docetaxel	¥39.86	128 mg	1.33	¥6,776.20	Zeng et al 2012 (17)
Pemetrexed	¥26.79	500 mg	1.33	¥17,861.33	Gu et al 2019 (18)
**Chemotherapy administration**					
Platinum-based	¥596.74/day		1.33		Zeng et al 2012 (17)
Single drug	¥270.87/day		1.33		Zeng et al 2012 (17)
**Management**					
Outpatient consult	¥382.68	–	1	¥382.68	Zeng et al 2012 (17)
CT	¥484.92	–	0.5	¥242.46	Zeng et al 2012 (17)
MRI	¥1101.34	–	0.5	¥550.67	Zeng et al 2012 (17)
Ultrasound	¥402.73	–	0.5	¥201.37	Zeng et al 2012 (17)
Best support care	¥1902.33	–	1	¥1902.33	Zeng et al 2012 (17)
Terminal care	¥17,423.00	–	1	¥17,423.00	Lu S et al, 2017 (19)

^*^77% of patients received platinum-based chemotherapy; ^^^23% of patients received pemetrexed.

### Costs

Since the healthcare system perspective was adopted to measure the cost and benefits, only direct medical costs were considered in the modelled economic analysis. Primary cost components included first-line treatment (drug acquisition and administration cost relating to dacomitinib and gefitinib), second- and third-line treatment, outpatient visit, and costs due to adverse events. The costs related to the treatment of commonly reported adverse events are included: for example, diarrhoea (56%), alanine aminotransferase increase (39%), and aspartate aminotransferase increase (36%) (details summarised in **Supplementary Table 2**). A 28-day cycle was adopted to estimate the costs according to the treatment regimen. The costs are expressed in Chinese yuan (CNY) valued in the year 2018. EGFR-TKIs drug cost used in the model is the national reimbursement price. All the unit costs of treatment are listed in **Table 1**.

### Utility Weights

The utility weights associated with being in the PF health states were sourced from the pivotal trial. For the PF state, patients who received dacomitinib (0.783) reported lower quality of life compared to those who were treated with gefitinib (0.828); using this, differentiated utility weights by treatment status are considered not favouring the intervention. Different utility weights were assigned for patients receiving second- or third-line TKI treatment, chemotherapy, or best supportive care to account for the different profiles associated with treatment-related adverse events post-progression. The utility weights applied in the modelled economic analysis are outlined in **Supplementary Table 3**.

### Cost-Effectiveness Analysis

The primary outcome measure was the quality-adjusted life year (QALY), which combines morbidity and mortality. Gefitinib was selected as the sole comparator since it has been reimbursed in China and adopted as the comparator for the economic evaluation of osimertinib in Australia that underpinned the reimbursement decision-making (18). In addition, there was no significant difference in effectiveness between erlotinib and gefitinib (and other first-line EGFR TKIs (19)). The incremental cost-effectiveness ratio (ICER) was calculated as the ratio between the incremental costs and incremental QALYs. All the costs and QALYs were accrued over a 15-year time period, given the relatively poor prognosis of the modelled population. In the absence of an official willingness-to-pay (WTP) per QALY threshold in China, three times the Gross Domestic Production (GDP) per capita (CNY 64,644×3) (20) from 2018 was adopted to examine the cost-effectiveness of dacomitinib. All the costs and benefits were discounted at a 5% rate per year (21).

### Sensitivity Analysis

Deterministic and probabilistic sensitivity analyses (DSA and PSA) were undertaken to test the robustness of base care results. In the DSA, a series of one-way sensitivity analyses were performed to examine the variation in ICER by varying one key parameter within a range at a time. The results were presented in the form of a Tornado diagram. In the PSA, the distribution of key uncertainty parameters was incorporated. The second-order Monte Carlo simulation technique was adopted to sample 1000 iterations from each distribution to parameterise the model and calculate the average across these 1000 iterations (and the 95% confidence interval). The results from the PSA were plotted in the incremental cost-effectiveness plane. The parameters that varied in the DSA and PSA are shown in **Supplementary Table 4**. Further, the various WTP/QALY thresholds were tested.

## Results

### Long-Term Extrapolation

In consultation with clinical experts and the AIC/BIC values and visual inspection, for PFS, Weibull and generalised gamma distribution were considered most plausible, while for OS, Weibull, Gompertz, and generalised gamma distribution were deemed reasonable. Following the NICE DSU recommendations, the same type of distribution for both arms of each endpoint is preferred. The different distributions have differential tail characteristics and therefore, utilising the same distribution could potentially avoid bias in the comparison generated by these differences. Moreover, the two treatment modalities compared are both TKIs, which have a similar mode of action. Hence, the Weibull distribution was chosen to extrapolate the PFS and OS curve regardless of treatment groups. Extrapolation of PFS and OS curves by alternative parametric survival functions is shown in **Supplementary Figures 1, 2**.

Published economic evaluations for similar EGFR TKIs were also reviewed. In the CEA of afatinib vs. gefitinib by Chouaid et al. (2017) (based on the LUX-Lung 7 trial), the authors used the Weibull distribution for both PFS and OS based on the AIC (22). Gefitinib was a common comparator between the LUX-Lung 7 and ARCHER 1050 trials, and afatinib and dacomitinib have analogous mechanisms of action. As a result, it is reasonable to assume that their long-term survival curves would follow a similar distribution. It is acknowledged that the LUX-Lung 7 trial had a complete follow-up period (i.e., 27.3 months) (23), which is more informative for model selection in lieu of long-term extrapolation. It is considered as an external data point justifying the selection of the Weibull distribution in the current economic evaluation.

**Figure 1:**
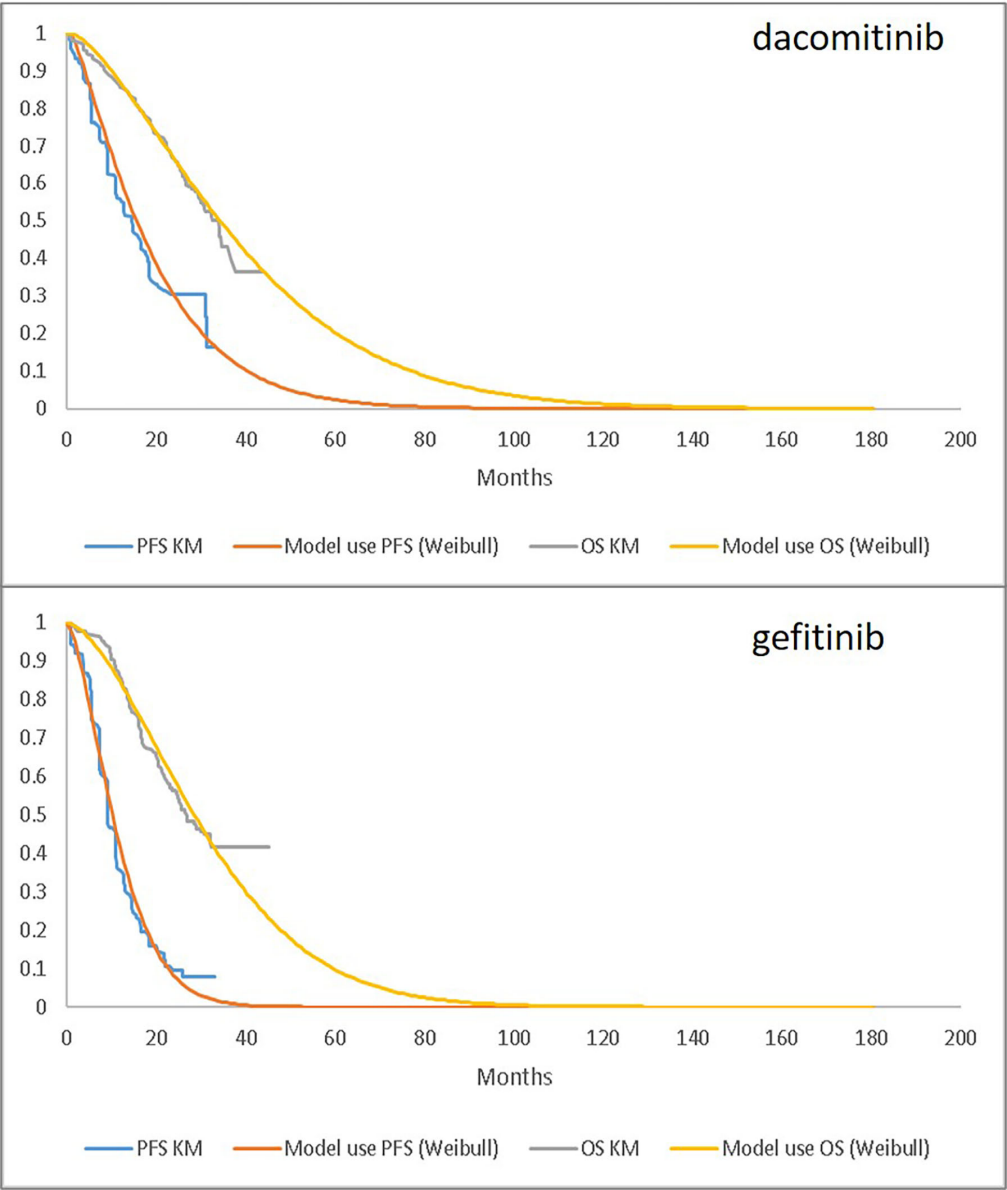
Parametric Fitting (Weibull) Compared to Observed KM Data: PFS (based on independent review committee) and OS for dacomitinib (upper) and gefitinib (lower). PFS, progression-free survival; OS, overall survival; KM, Kaplan-Meier.

The goodness-of-fit statistics for fitting PFS and OS curves are provided in **Table 2**, and fitted parametric curves are shown in **Figure 1**. The PFS results based on the independent review committee (IRC) were used for the modelled cost-effectiveness analysis.

**Table 2:** Goodness-of-Fit Statistics for the PFS and OS by treatment groups.

Treatment	Curve (Weibull)	AIC	BIC	Mean (month)	Median (month)
Dacomitinib	PFS (IRC)	545.20	552.04	18.67	14.74
	PFS (INV)	530.36	537.21	19.06	15.70
	OS	465.03	471.88	38.92	33.36
Gefitinib	PFS (IRC)	514.46	521.29	11.80	10.25
	PFS (INV)	513.42	520.25	13.25	11.60
	OS	461.29	468.12	32.01	28.55

AIC, Akaike information criterion; BIC, Bayesian information criterion; IRC, independent review committee; INV, investigator; OS, overall survival.

### Cost-Effectiveness Analysis

Over a 15-year time period, dacomitinib (CNY 265,512 and 1.96 QALY) was associated with higher costs and QALY gains compared to gefitinib (CNY 247,048 and 1.61 QALY), resulting in an ICER of CNY 58,947/QALY. Using the empirical WTP/QALY threshold, it is considered that dacomitinib is a cost-effective treatment strategy for patients with EGFR-mutation-positive advanced NSCLC. The key cost components included costs related to first-line medications (CNY 108,795 and 83,414), outpatient care (CNY 81,944 and 73,215), second- and third-line medications (CNY 59,446 and 74,699), terminal care (CNY15,290 and 15,690), and AE (CNY 37 and 30) in dacomitinib and gefitinib groups, respectively (**Table 3**).

**Table 3:** Base case results of the cost-effectiveness analysis.

Treatment	Cost (CNY)	QALYs	Incremental Cost (CNY)	Incremental QALYs	ICER
Dacomitinib	265,512	1.9548			
Gefitinib	247,048	1.6067	18,463	0.3132	58,947

CNY, Chinese Yuan; QALY, quality-adjusted life year; ICER, incremental cost-effectiveness ratio.

### Sensitivity Analyses

**Figure 2:**
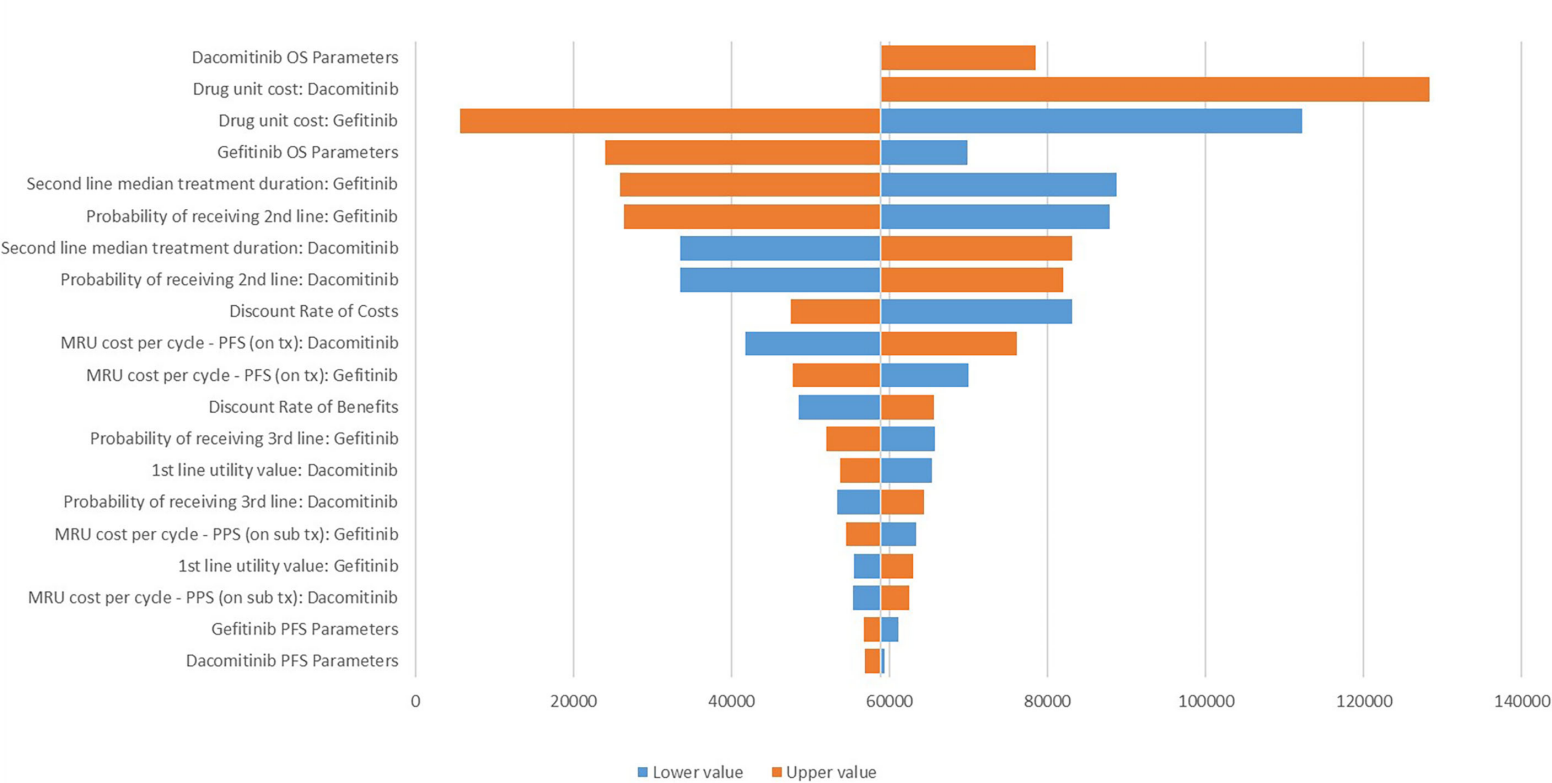
Tornado diagram for the one-way sensitivity analysis. MRU, medical resource use; tx, treatment; OS, overall survival; PFS, progression-free survival; PPS, post-progression survival. The lower values were not tested for the dacomitinib OS parameters and its unit cost due to the negative ICER generated.

The DSA identified that drug acquisition cost for dacomitinib and gefitinib, dacomitinib OS extrapolation, second-line treatment duration and probability of receiving second-line treatment post-gefitinib, and second-line treatment duration post-dacomitinib are the key determinants for the ICER. At the same time, probability of receiving third-line treatment post-dacomitinib, the medical resource use (i.e., outpatient care) cost per cycle for both dacomitinib and gefitinib, and gefitinib/dacomitinib PFS extrapolation are less determinant for the ICER (**Figure 2**).

The base case ICER was moderately sensitive to the parametric survival distributions adopted (i.e., for extrapolating survival curves for gefitinib); for example, if the generalised gamma distribution was selected for the PFS curve while Gompertz distribution was used for the OS curve, the ICER increased to CNY 70,152/QALY (**Supplementary Table 4**).

**Figure 3:**
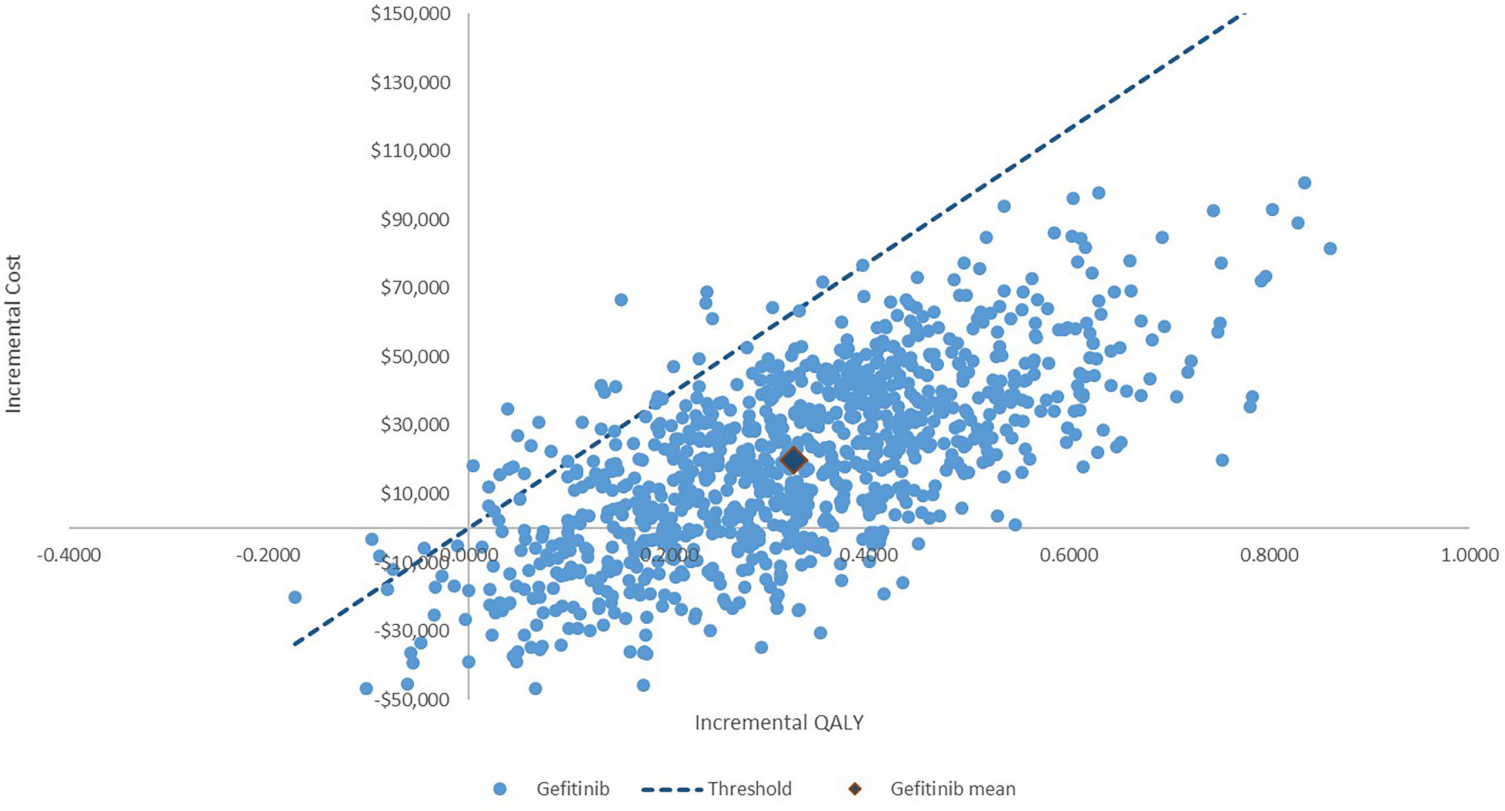
Incremental cost-effectiveness plane: Dacomitinib vs. gefitinib. The probability of dacomitinib being cost-effective is 97%.

**Figure 4:**
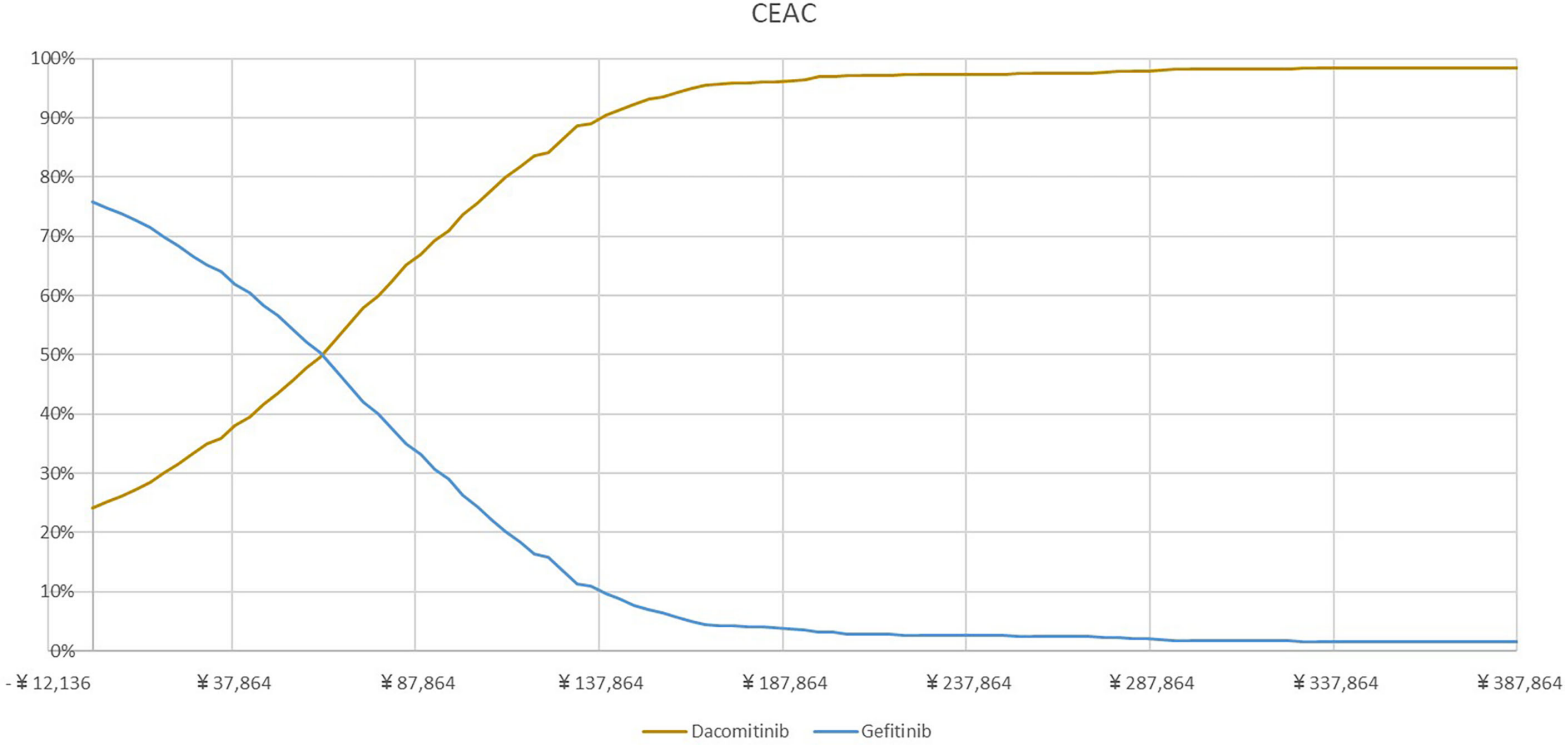
Cost-effectiveness acceptability curve.

The PSA showed that most of the results demonstrated that dacomitinib contributed to greater costs and QALYs, suggesting a probability of 97% of being cost-effective compared to gefitinib (**Figure 3**). The cost-effective acceptability curve is shown in **Figure 4**, which shows that when the WTP/QALY was over three times the GDP/Capita in China, dacomitinib becomes highly likely to be cost-effective (over 90%). Lowering the WTP/QALY to two or one times the GDP/Capita and reducing cost-effective probability to 89% and 54%, respectively (**Supplementary Figures 3, 4**).

## Discussion

The modelled cost-effectiveness analysis of dacomitinib as a first-line treatment for patients with locally advanced or metastatic EGFR-mutation-positive NSCLC in China was associated with an ICER of CNY 58,947/QALY compared with gefitinib over a 15-year time period. The incremental cost and QALYs were CNY 18,463 and 0.3132, respectively. Using the empirical WTP/QALY threshold in China, dacomitinib is considered a cost-effective treatment modality in this population from the Chinese healthcare payer’s perspective.

It is acknowledged that the parametric survival models for long-term extrapolation play a key role in determining the cost-effectiveness of the intervention. Sensitivity analyses were thus undertaken by varying the model parameters and testing the alternative distributions (i.e., generalised gamma). Not surprisingly, the sensitivity analyses indicated that the OS parameters for dacomitinib and gefitinib were critical drivers for the ICER. The sensitivity analyses by adopting alternative parametric distribution showed that even after adopting alternative distribution to extrapolate the within-trial observation, dacomitinib was still a cost-effective treatment strategy compared to gefitinib.

The patient-level data were utilised to derive the long-term extrapolation, which captured all the possible covariates that might have influenced the OS and PFS over the trial duration and reflected the time dependence. The PartSA approach directly applies the primary outcomes from the pivotal trial (i.e., PFS and OS) and derivation of the state membership from the survival function directly. As the OS curve was utilised directly to estimate the proportion of patients in the death state over time, the OS from the PartSA was a perfect match to the observed OS within-trial in this approach. In a 2017 NICE DSU review of NICE oncology technology appraisals, 73% (22/30) of the appraisal for cancer interventions adopted the PartSA to assess the long-term cost-effectiveness of the intervention. It is believed that this is an appropriate modelling technique in this case as well.

Of particular importance, the base case cost-effectiveness results were based on the PFS outcome assessed by the independent review committee, which is considered conservative. The pivotal trial showed median PFS was 14.7 (95%CI: 11.1-16.6) vs 9.2 months (95%CI: 9.1-11.0) from the independent review committee (HR 0.59, 95% CI: 0.47-0.74), while the same outcome was 16.6 (95%CI: 12.9-18.4) vs 11.0 months (95%CI: 9.4-12.1) from the investigators’ judgement (HR 0.62, 95%CI: 0.50-0.78).

The QALY outcome of dacomitinib from the current study (i.e., when a 10-year time period was adopted, the QALY gain was 1.938 and 1.629 in dacomitinib and gefitinib groups) was similar to other published cost-effectiveness analyses concerning similar therapies. The modelled economic analysis of afatinib versus gefitinib reported a QALY of 1.857 and 1.687, respectively, which also extrapolated the OS and PFS curves using the Weibull distribution whereas they adopted a shorter time frame (i.e., 10 years) and a 4% discount rate (22). Another report had slightly lower QALY gains for the assessed TKIs conducted in the United States (i.e., 1.50 QALY for afatinib, 1.51 QALY for erlotinib, and 1.47 QALY for gefitinib), compared with the current results. However, since the US study did not have access to the individual-level patient data, the long-term extrapolation may be less accurate than this presented study which extrapolated the within-trial data based on individual patient data. In terms of the incremental costs, the previous studies reported €7,700 and $7,714 respectively in the base case scenarios. With the simple currency conversion, incremental costs were similar across these modelled economic evaluations. Another economic analysis that compared afatinib with pemetrexed-cisplatin in the same population reported an ICER of SG$137,648/QALY (24) (the QALY gain was 1.69 in the afatinib treatment group) based on the PartSA technique. A French study compared afatinib versus erlotinib as a second-line treatment (patient failed platinum-based therapy) for NSCLC, the QALY gain was lower than those in the first-line treatment setting (0.94 versus 0.78 in these patients with more advanced disease), but concluded it was highly likely to be cost-effective over a 10-year time period with a corresponding ICER of €30,277/QALY (25).

The empirical WTP/QALY is established using the WHO recommendation of one to three times of GDP/Capita, and we also examined the cost-effectiveness conclusion by varying such a threshold in the sensitivity analyses. Three times the GDP/Capita is usually adopted for non-developed countries, and this threshold is consistent with prior published economic evaluation in China (26–28).

This study is not without limitations. First, only the PartSA modelling technique was utilised to simulate the long-term costs and QALY associated with dacomitinib treatment. Because the primary assumption underlying the PartSA approach (i.e., PFS and OS are independent, so PFS is not predictive of OS), this assumption cannot hold sometimes. Second, the treatment with dacomitinib/gefitinib was discontinued upon disease progression in the model; however, in actual clinical practice, patients may continue such treatment with the treating physician’s discretion. Third, the patients recruited in the trial may not be the same as the characteristics of patients in China. For example, ARCHER 1050 had more women and non-smokers and patients with less advanced NSCLC compared to the real-world patients (29). Therefore, the cost-effectiveness analysis based on the trial population may not be directly applicable for the Chinese patient population (30). Nevertheless, this is the first published economic evaluation of dacomitinib in treating patients with EGFR-mutation-positive NSCLC, which was performed based on the individual patient data that can maximise the accuracy of the long-term extrapolation for the OS and PFS curves, which bears important implications for policy decision-making. The economic evaluation was performed from the Chinese healthcare system perspective; however, the results may be helpful for other countries with similar economic status.

## Conclusions

Dacomitinib is a cost-effective treatment strategy in the first-line treatment of patients with EGFR-mutation-positive NSCLC from the Chinese healthcare payer’s perspective. The uncertainty around the cost-effectiveness of dacomitinib could be reduced if long-term survival data become available.

## Data Availability Statement

The data analyzed in this study is subject to the following licenses/restrictions: The data used for the current study can be requested on a reasonable basis. Requests to access these datasets should be directed to shunlu@sjtu.edu.cn.

## Ethics Statement

The studies involving human participants were reviewed and approved by the institutional review board of Shanghai Chest Hospital, Shanghai, China. The patients/participants provided their written informed consent to participate in this study.

## Author Contributions

Y-FY, LL, F-FZ, PD, L-HM, L-TL, LG, and SL contributed to the conception and design of the study. LL undertook the analysis. All the authors contributed to the result interpretation and critically reviewed the manuscript.

## Funding

This work was funded by the National Key R&D Program of China (2016YFC1303300), the National Natural Science Foundation of China (81672272), and Shanghai Municipal Science & Technology Commission Research Project (17431906103).

## Conflict of Interest

LL, F-FZ, PD, and L-HM are employees of Pfizer China. L-TL was employed by Shanghai PalanDataRx Co., Ltd.

The remaining authors declare that the research was conducted in the absence of any commercial or financial relationships that could be construed as a potential conflict of interest.

## Publisher’s Note

All claims expressed in this article are solely those of the authors and do not necessarily represent those of their affiliated organizations, or those of the publisher, the editors and the reviewers. Any product that may be evaluated in this article, or claim that may be made by its manufacturer, is not guaranteed or endorsed by the publisher.
