# Supplementary material for: Modelled Economic Analysis for Dacomitinib–A Cost Effectiveness Analysis in Treating Patients With EGFR-Mutation-Positive Non-Small Cell Lung Cancer in China
Source: Front Oncol. 2021 Dec 14;11:564234. doi: 10.3389/fonc.2021.564234 (PMC8712321; doi:10.3389/fonc.2021.564234)
Supplement: Supplementary file 1 [file DataSheet_1.docx]

# **Supplementary document**

Supplementary Figure 1. Long-term Extrapolation of PFS (A) and OS (B) for Dacomitinib

**A**


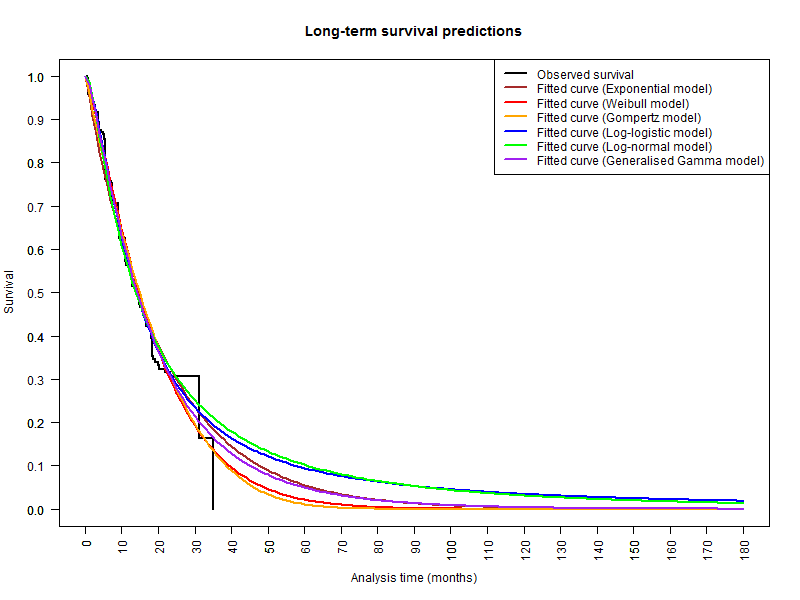


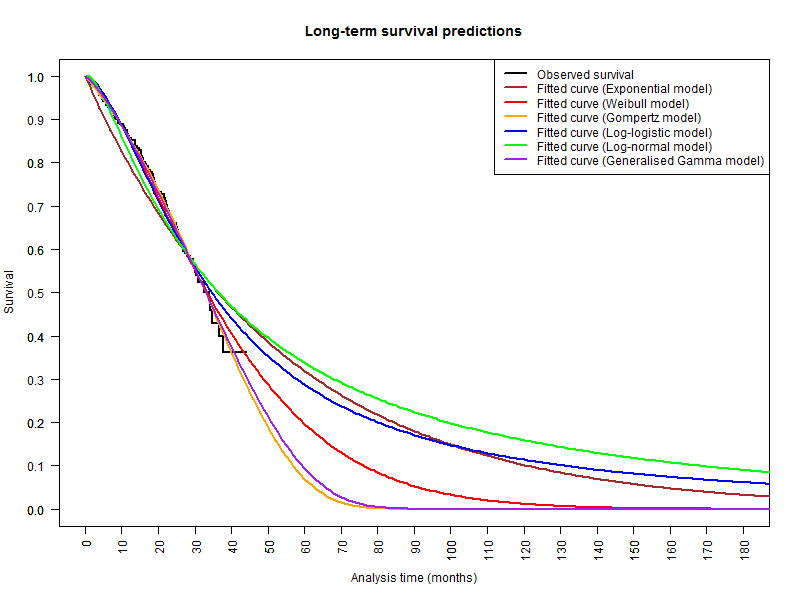


**B**

Supplementary Figure
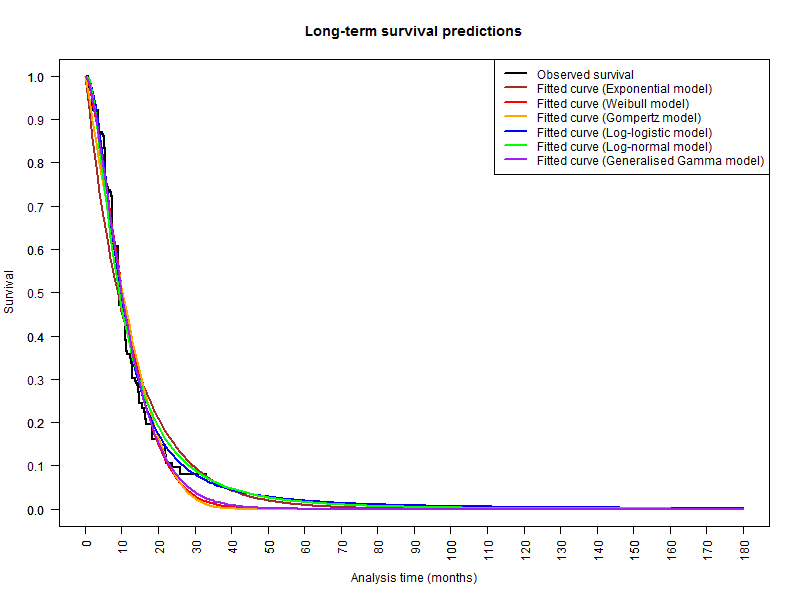
2. Long-term extrapolation of PFS (A) and OS (B) for Gefitinib

**A**


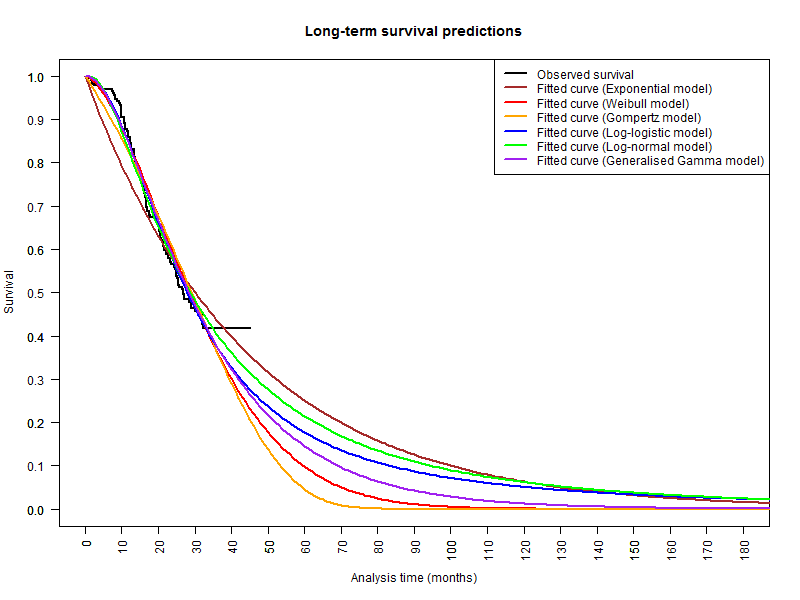


**B**

## **Supplementary Figure 3. Incremental cost-effectiveness plane_two time of GDP/Capita as WTP/QALY threshold**


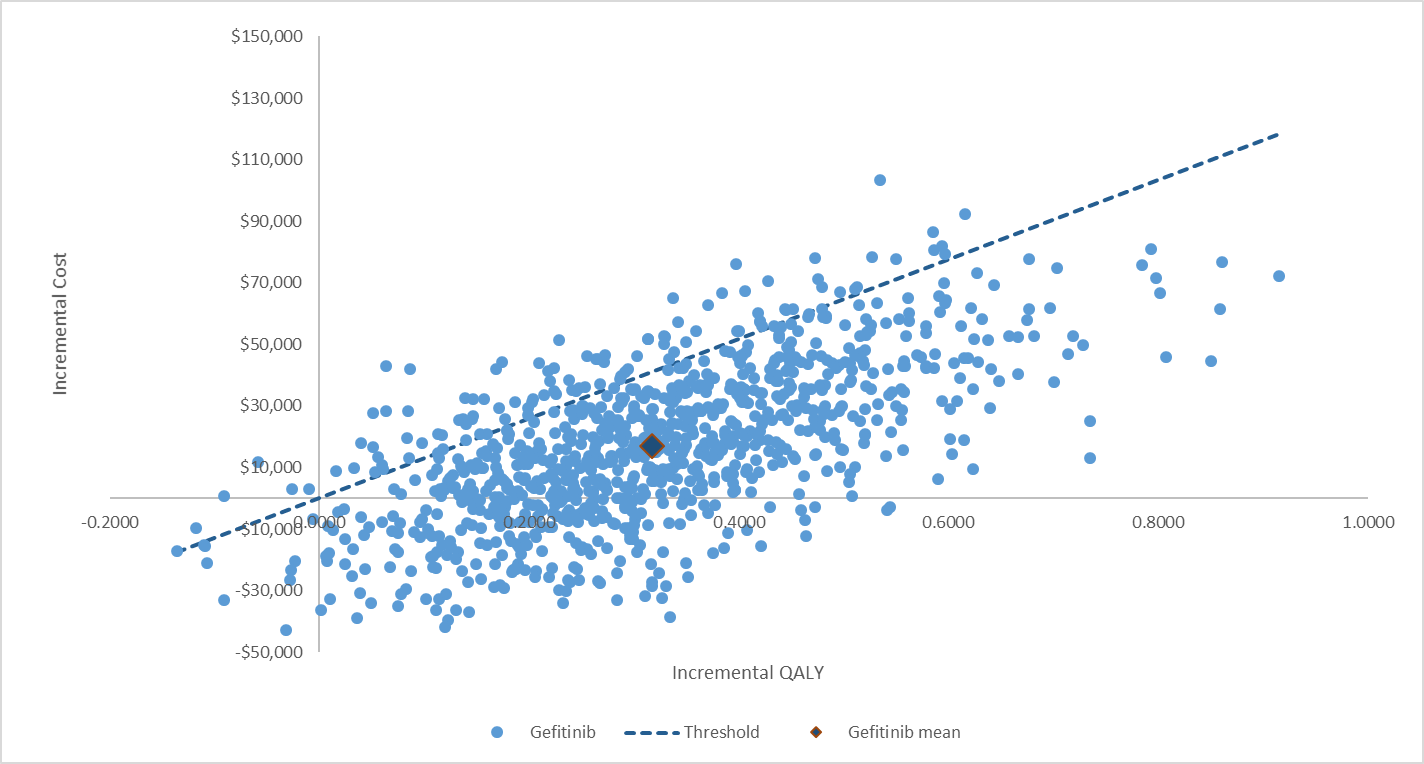


## **Supplementary Figure 4. Incremental cost-effectiveness plane_one time of GDP/Capita as WTP/QALY threshold**


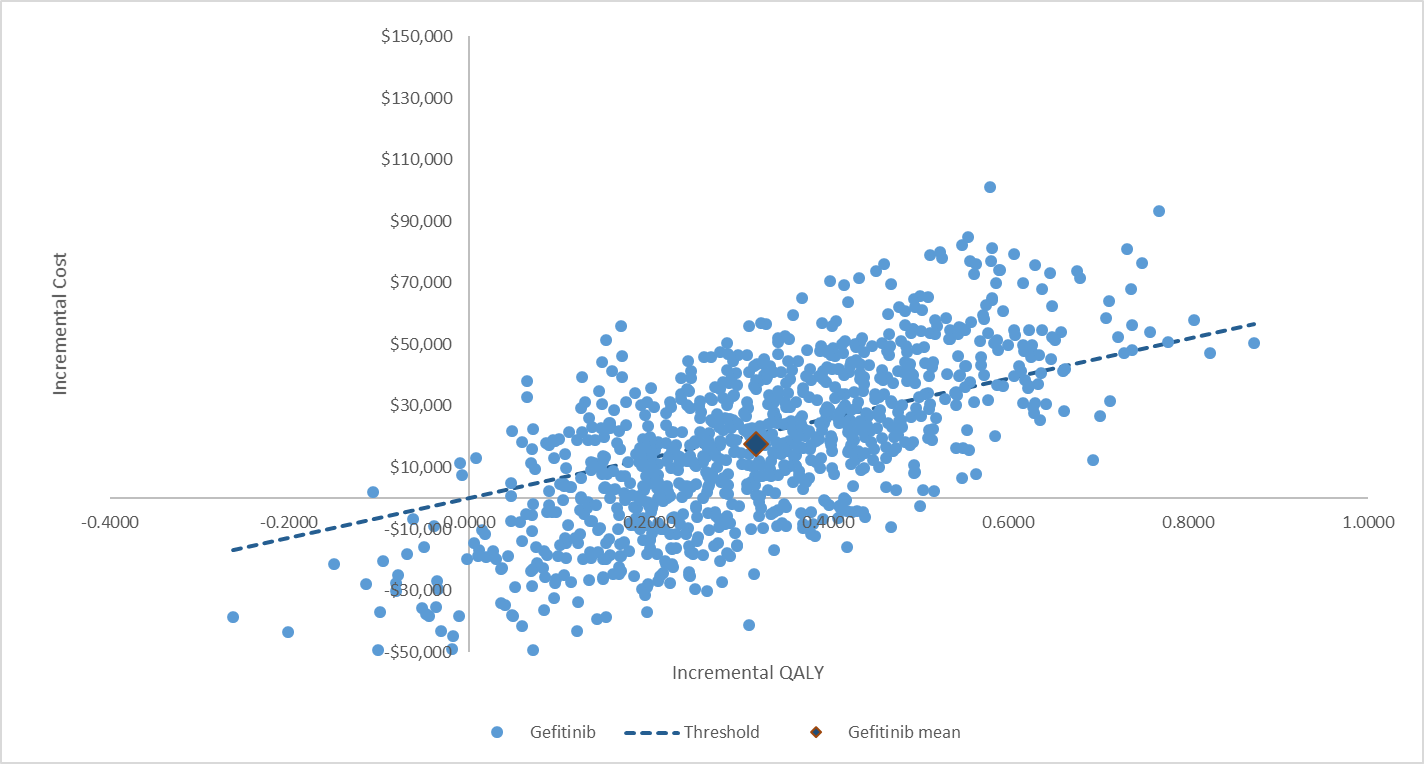


## **Supplementary Table 1. Details for the second- and third-line treatment**

|  | **Proportion** | | **Treatment duration(cycle)** | |
| --- | --- | --- | --- | --- |
|  | **dacomitinib** | **gefitinib** | **dacomitinib** | **gefitinib** |
| **Second-line** | | | | |
| Afatinib |  | 5% |  | 6.20 |
| Gefitinib | 5% |  | 6.20 |  |
| Erlotinib | 5% | 5% | 6.20 | 6.20 |
| Osimertinib | 50% | 50% | 11.96 | 11.96 |
| Platinum-based chemotherapy | 40% | 40% | 3.15 | 3.15 |
| Single drug chemotherapy | 0 | 0 |  |  |
| **Third-line** | | | | |
| Afatinib |  | 5% |  | 3.15 |
| Gefitinib | 5% |  | 3.15 |  |
| Erlotinib | 5% | 5% | 3.15 | 3.15 |
| Osimertinib | 10% | 10% | 11.96 | 11.96 |
| Platinum-based chemotherapy | 45% | 45% | 2.72 | 2.72 |
| Single drug chemotherapy | 35% | 35% | 2.72 | 2.72 |

## **Supplementary Table 2 Details for the treatment-related adverse events**

| **Adverse events** |  | Dacomitinib (%) | Gefitinib (%) |
| --- | --- | --- | --- |
| Diarrhoea | ¥45.27 | 8.8% | 0.9% |
| Paronychia | ¥352.90 | 7.5% | 1.3% |
| Acne-like dermatitis | ¥5.82 | 13.7% | 0.0% |
| Oral mucositis | ¥9.79 | 3.5% | 0.4% |
| Loss of appetite | ¥101.43 | 3.1% | 0.4% |
| Weight loss | ¥0 | 2.2% | 0.4% |
| Increased ALT | ¥295.84 | 0.9% | 8.5% |
| Rash | ¥10.89 | 4.4% | 0.0% |

ALT: alanine aminotransferase

### **Supplementary Table 3 Utility weights used in the cost-utility analysis**

| **Health state** | **Utility** | **Reference** |
| --- | --- | --- |
| Progression- free with dacomitinib | 0.780 | Wu et al 2017^9^ |
| Progression- free with gefitinib | 0.828 | Wu et al 2017 |
| Progressed with second-line TKI treatment | 0.805 | Bertranou et al 2018^26^ |
| Progressed with second-line chemotherapy | 0.778 | Bertranou et al 2018 |
| Progressed with third-line TKI treatment | 0.62 | Chouaid et al 2013^27^ |
| Progressed with third-line chemotherapy | 0.62 | Chouaid et al 2013 |
| Progressed with best-support care | 0.47 | Nafees et al 2008^28^ |

## **Supplementary Table 4. Parameters tested in the sensitivity analysis**

| **Variable** | **Base care** | **Lower bound** | **Upper bound** |
| --- | --- | --- | --- |
| Discount Rate of Costs | 5% | 0% | 8.0% |
| Discount Rate of Benefits | 5% | 0% | 8.0% |
| Time horizon | 15 years | 5 | 20 |
| Dacomitinib OS Parameters | From parametric survival analysis | Based on Cholesky decomposition | Based on Cholesky decomposition |
| Gefitinib OS Parameters |  |  |  |
| Dacomitinib PFS Parameters |  |  |  |
| Gefitinib PFS Parameters |  |  |  |
| 1st line utility value: Dacomitinib | 0.78 | 0.76 | 0.80 |
| 1st line utility value: Gefitinib | 0.83 | 0.81 | 0.85 |
| Probability of receiving 2nd line: Dacomitinib | 71% | 0.57 | 0.85 |
| Probability of receiving 2nd line: Gefitinib | 71% | 0.57 | 0.85 |
| Probability of receiving 3rd line: Dacomitinib | 48% | 0.38 | 0.58 |
| Probability of receiving 3rd line: Gefitinib | 48% | 0.38 | 0.58 |
| Second line median treatment duration: Dacomitinib | 5.41 | 4.33 | 6.49 |
| Second line median treatment duration: Gefitinib | 5.41 | 4.33 | 6.49 |
| Third line median treatment duration: Dacomitinib | 2.99 | 2.39 | 3.59 |
| Third line median treatment duration: Gefitinib | 2.99 | 2.39 | 3.59 |
| Drug unit cost per cycle: Dacomitinib | 5437.34 | 4349.90 | 6524.85 |
| Drug unit cost per cycle: Gefitinib | 6343.68 | 5074.94 | 7612.42 |
| MRU cost per cycle - PFS (on tx): Dacomitinib | 208.12 | 166.49 | 249.74 |
| MRU cost per cycle - PFS (on tx): Gefitinib | 208.12 | 166.49 | 249.74 |
| MRU cost per cycle - PPS (on 1st line): Dacomitinib | 208.12 | 166.49 | 249.74 |
| MRU cost per cycle - PPS (on 1st line): Gefitinib | 208.12 | 166.49 | 249.74 |
| MRU cost per cycle - PPS (on sub tx): Dacomitinib | 208.12 | 166.49 | 249.74 |
| MRU cost per cycle - PPS (on sub tx): Gefitinib | 208.12 | 166.49 | 249.74 |
| AE cost per event: Dacomitinib | 5.57 | 4.46 | 6.69 |
| AE cost per event: Gefitinib | 4.57 | 3.66 | 5.49 |

## **Supplementary Table 4a. Additional results from the deterministic sensitivity analysis**

| Scenario Description | Base-Case Scenario | Alternative Scenarios | ICER/QALY |
| --- | --- | --- | --- |
| **Base case** | | | CNY 58,947 |
| General Model Settings | | | |
| Time horizon | 15 years | 5 years | CNY 37,008 |
|  |  | 10 years | CNY 58,127 |
|  |  | 20 years | CNY 58,948 |
| Discount rate | 5% | 0% | CNY 68,507 |
|  |  | 8% | CNY 52,898 |
| Clinical Efficacy Approach | | | |
| Gefitinib OS projection | PFS: Weibull  OS: Weibull | OS:Gompertz | CNY 68,928 |
| Gefitinib PFS projection | PFS:Weibull  OS: Weibull | PFS: Generalized Gamma | CNY 60,011 |
| Gefitinib PFS and OS projection | PFS: Weibull  OS: Weibull | PFS: Generalized Gamma  OS: Gompertz | CNY 70,152 |
| **Cost Settings** | | | |
| Cost category | All healthcare costs^*^ | Drug cost only | CNY 81,056 |

*including drug costs, disease management costs, subsequent treatment costs, terminal costs

Abbreviations: ICER: incremental cost-effectiveness ratio; QALY: quality-adjusted life year; PFS: progression-free survival; OS: overall survival; CNY: Chinese yuan
